# Evaluation of Calyculin A Effect on γH2AX/53BP1 Focus Formation and Apoptosis in Human Umbilical Cord Blood Lymphocytes

**DOI:** 10.3390/ijms22115470

**Published:** 2021-05-22

**Authors:** Lucián Zastko, Anna Račková, Petra Petrovičová, Matúš Durdík, Jakub Míšek, Eva Marková, Igor Belyaev

**Affiliations:** 1 Department of Radiobiology, Cancer Research Institute, Biomedical Research Center, Slovak Academy of Sciences, University Science Park for Biomedicine, Dúbravská Cesta 9, 845 05 Bratislava, Slovakia; ankasumi@gmail.com (A.R.); exonpetr@savba.sk (P.P.); exondurd@savba.sk (M.D.); exonfare@savba.sk (E.M.); igor.beliaev@savba.sk (I.B.); 2 Department of Medical Biophysics, Jessenius Faculty of Medicine in Martin, Comenius University in Bratislava, Malá Hora 4, 036 01 Martin, Slovakia; jakub.misek@jfmed.uniba.sk

**Keywords:** apoptosis, calyculin A, DMSO, DNA double-strand breaks, DNA repair foci, human umbilical cord blood lymphocytes, γH2AX pan-staining, γ-radiation

## Abstract

Dephosphorylation inhibitor calyculin A (cal A) has been reported to inhibit the disappearance of radiation-induced γH2AX DNA repair foci in human lymphocytes. However, other studies reported no change in the kinetics of γH2AX focus induction and loss in irradiated cells. While apoptosis might interplay with the kinetics of focus formation, it was not followed in irradiated cells along with DNA repair foci. Thus, to validate plausible explanations for significant variability in outputs of these studies, we evaluated the effect of cal A (1 and 10 nM) on γH2AX/53BP1 DNA repair foci and apoptosis in irradiated (1, 5, 10, and 100 cGy) human umbilical cord blood lymphocytes (UCBL) using automated fluorescence microscopy and annexin V-FITC/propidium iodide assay/γH2AX pan-staining, respectively. No effect of cal A on γH2AX and colocalized γH2AX/53BP1 foci induced by low doses (≤10 cGy) of γ-rays was observed. Moreover, 10 nM cal A treatment decreased the number of all types of DNA repair foci induced by 100 cGy irradiation. 10 nM cal A treatment induced apoptosis already at 2 h of treatment, independently from the delivered dose. Apoptosis was also detected in UCBL treated with lower cal A concentration, 1 nM, at longer cell incubation, 20 and 44 h. Our data suggest that apoptosis triggered by cal A in UCBL may underlie the failure of cal A to maintain radiation-induced γH2AX foci. All DSB molecular markers used in this study responded linearly to low-dose irradiation. Therefore, their combination may represent a strong biodosimetry tool for estimation of radiation response to low doses. Assessment of colocalized γH2AX/53BP1 improved the threshold of low dose detection.

## 1. Introduction

DNA double-strand breaks (DSB) are considered the most critical lesions since they can cause cell death and genetic instability [1]. Mis-rejoined DSB might lead to mutations and may, therefore, represent the initiating event for carcinogenesis. Phosphorylation of the histone variant 2AX (H2AX) at serine 139 is among the early steps in cell responses to DSB. Thousands of H2AX molecules contiguous to the DSB become phosphorylated within a few minutes after the formation of a DSB [2]. By courtesy of a specific antibody against the phosphorylated H2AX (so-called γH2AX) and a fluorescent secondary antibody, distinct DNA repair foci representing DSB can be detected within the cell nuclei by fluorescence microscopy [3]. Another useful molecular marker for DSB is a p53-binding protein 53BP1 [4,5], which is a well-known promoter of DSB repair by non-homologous end joining (NHEJ) [6,7,8]. Assessment of ionizing radiation-induced foci (IRIF) by immunostaining with antibodies to γH2AX/53BP1 has been shown to be a reliable and sensitive tool in the evaluation of DSB, including those which are induced in vivo by low dose ionizing radiation during radiologic procedures like computed tomography (CT) and angiography [9,10,11,12,13,14]. For the radiation doses obtained from ordinary CT scans, which are less than 10 cGy, usage of DNA repair foci assay remains the only possibility for measuring DSB as far as other methods lack appropriate sensitivity [10,15]. Such measurements are of key importance for estimating cancer risks dealing with exposure to low-dose ionizing radiation. In particular, there have been several published studies suggesting an increased risk of leukemia and tumors, especially in children exposed to CT radiation of less than 10 cGy [16,17,18]. While the γH2AX and 53BP1 are commonly accepted molecular markers for biological dosimetry and enumeration of DSB, both γH2AX and 53BP1 foci disappear quickly due to γH2AX dephosphorylation and re-localization of 53BP1 from the location of the DSB [19]. The peak number of foci is usually observed at 15 min and gradually declines afterward [9]. This kinetics limits the number of samples that can be prepared simultaneously when a peak number of foci appears (limiting factors: number of microscopic slides to be managed at once and time of their preparation and centrifugation to fixation). Hence, the real number of DSB may be underestimated due to ongoing repair during the workup of multiple samples. To solve this problem, the methodological approach for analysis of peak number of γH2AX foci in multiple samples by stabilizing degradation of γH2AX foci via inhibiting its dephosphorylation using calyculin A (cal A) has been suggested [20].

Cal A is a well-known inhibitor of phosphatase 1 and 2A. It has been reported to effectively inhibit γH2AX dephosphorylation in cell cultures after irradiation with higher doses of γ-rays [21,22]. Also, cal A was reported to inhibit the disappearance of γH2AX foci in human lymphocytes with doses as low as commonly used in diagnostic and interventional radiology [20]. However, other studies reported no effect of cal A on the kinetics of γH2AX focus induction and loss in irradiated cells [23,24]. The study by Kuefner et al. [20] has drawn our attention due to its potentially remarkable results for eliminating the influence of DNA repair on the assessment of low dose effects by γH2AX. This result was strengthened by the finding that cal A did not affect the yield of 53BP1 foci as far as formation and loss of 53BP1 is not expected to depend on phosphorylation [20]. However, Kuefner et al. did not investigate the effect of cal A on co-localization of γH2AX and 53BP1 foci, which is considered to be more specific DSB marker than γH2AX or 53BP1 foci evaluated individually. Furthermore, as authors themselves stated they had to use other cells than lymphocytes (fibroblasts) to test the toxicity of cal A by colony formation assay (CFA) and therefore suggested that the results of CFA were not directly transferable to lymphocytes. Thus, to determine the survival of the cal A treated lymphocytes, we decided to analyze apoptosis in the same cal A treated samples of human umbilical cord blood lymphocytes (UCBL) as were used for foci evaluation.

As long as cal A might be a valuable tool in the estimation of DSB induction caused directly/indirectly by radiation using therapeutic or diagnostic procedures, we aimed in this study to analyze the effect of cal A on the number of γH2AX and 53BP1 foci and their colocalization in lymphocytes after in vitro irradiation with low and high doses of γ-rays. In accordance with previously published data of Kuefner et al. [20] and Roch-Lefèvre [24], we have chosen to investigate two cal A concentrations of 1 and 10 nM since they have been known to stabilize γH2AX foci levels for 2 h in human lymphocytes. We also followed the kinetics of apoptosis with annexin V-FITC/propidium iodide (PI) assay 2, 20 and 44 h post-irradiation in cal A treated cells. In addition, we used γH2AX pan-staining assay, which is a recognized marker for early apoptosis [25,26,27,28].

## 2. Results

### 2.1. Overall, Assessment of Data at 2 h Post-Irradiation

We studied DNA repair foci and apoptosis induced by cal A in UCBL of three different probands. Considering all endpoints (levels of γH2AX, 53BP1, and colocalized γH2AX/53BP1 foci, ratios of viable, early apoptotic (EA) and late apoptotic/necrotic (LAN) cells and percentage of γH2AX pan-stained cells), multivariate ANOVA has revealed a significant effect of type of treatment (that includes both cal A treatments, DMSO treatment and untreated cells) (*p* = 0.00000) and delivered irradiation dose (*p* = 0.00000) 2 h post-irradiation (Table 1). Univariate analysis for each dependent variable has shown that not all of the followed endpoints responded equally to the type of treatment and irradiation dose (Table 1). While the number of foci consistently depended on irradiation dose, no dose dependence of all apoptotic endpoints was revealed at 2 h post-irradiation (Table 1). Reversely, all of the followed endpoints (besides γH2AX foci formation) were significantly affected by treatment type. When all endpoints (apoptosis/foci) were considered 2 h post-irradiation, their statistically significant dependence on used cal A concentration has been revealed (*p* = 0.02463).

### 2.2. Overall, Assessment of Apoptosis Measured at 2, 20 and 44 h Post-Irradiation

We have also investigated apoptosis in these differently treated and irradiated UCBL 20 and 44 h post-irradiation using flow cytometry and annexin V FITC/PI assay. All of pursued apoptotic endpoints (ratios of viable, EA and LAN cells) responded significantly to the treatment type (*p* = 0.00000), delivered irradiation dose (*p* = 0.00000) and post-irradiation time (*p* = 0.00000) (Table 2). This consistent pattern also has been seen in results from univariate statistical analysis for each dependent variable except for ratios of EA UCBL that seemed not to be affected by delivered doses considering all time points (Table 2). Furthermore, when all apoptotic endpoints were considered 2, 20 and 44 h post-irradiation, no statistically significant dependence on cal A concentration used occurred.

### 2.3. DNA Damage Response in UCBL Induced by Cal A 2 h Post-Irradiation

#### 2.3.1. DNA Repair Foci

It is well-known that the level of IRIF reaches its peak around 15 to 30 min post-irradiation and then declines due to DNA damage repair. In our experiment, UCBL were fixed 2 h post-irradiation providing sufficient time for a significant decrease of IRIF. Given the dephosphorylation inhibitory properties of cal A and activation of histone H2AX via phosphorylation, we expected to see distinct contrast between cal A treated and DMSO treated cells in the levels of γH2AX (possibly colocalized γH2AX/53BP1) foci 2 h post-irradiation. 53BP1 foci were not expected to be affected by cal A treatments.

##### Gamma-Radiation

**Figure 1:**
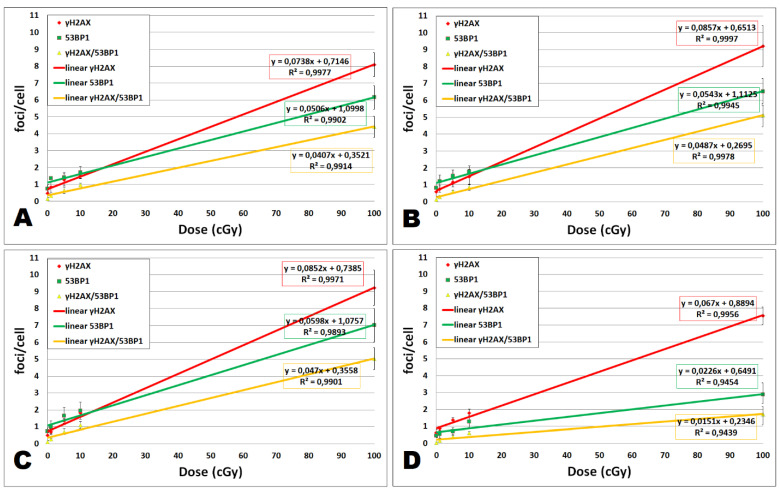
Linear fit for dose dependence of γH2AX, 53BP1, and colocalized γH2AX/53BP1 foci at 2 h post-irradiation. Dose dependence of focus induction and linear regression along with the coefficient of determination R^2^ is shown for different treatments: (**A**) untreated, (**B**) 0.1% DMSO, (**C**) cal A (1 nM), and (**D**) cal A (10 nM) treatment. Data from 3 experiments are shown for each dose (0, 1, 5, 10, and 100 cGy). Vertical bars represent standard deviations.

**Figure 2:**
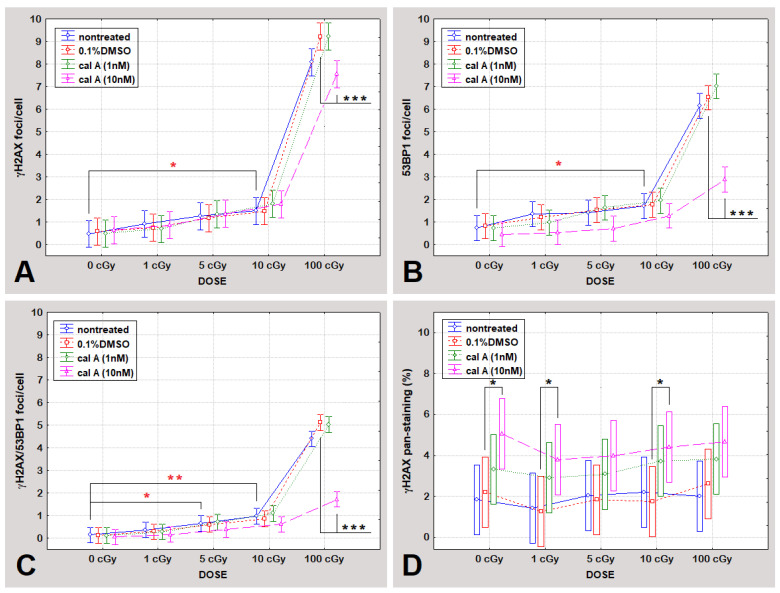
The level of γH2AX foci (**A**), 53BP1 foci (**B**), colocalized γH2AX/53BP1 foci (**C**), and γH2AX pan-staining (**D**) at 2 h post-irradiation. DNA repair foci levels and percentual rates of γH2AX pan-staining of differently treated cells (untreated, 0.1% DMSO, cal A (1 nM), and cal A (10 nM)) irradiated with 0, 1, 5, 10, and 100 cGy are shown. Each data point shows the mean value and ±0.95 confidence interval from 3 experiments. * indicates statistically significant difference between irradiated and unirradiated cells; * indicates statistically significant differences between treatments. * *p* ˂ 0.05, ** *p* ˂ 0.0099, ***p ˂ 0.00099.

As Figure 1A shows, all dose responses fit linear dependencies (R^2^ ≤ 0.9902). We have seen significant induction of colocalized γH2AX/53BP1 foci by the dose of 5 cGy (*p* = 0.043) in untreated UCBL, while γH2AX and 53BP1 foci enumerated separately were significantly induced by the dose of 10 cGy (*p* = 0.02 and 0.017, respectively) (Figure 2), confirming that colocalization of γH2AX/53BP1 foci being more sensitive radiation-induced DNA damage marker than when each type of foci evaluated individually. Thus, our assay has proven to be very sensitive and, therefore, eligible to uncover eventual effects of cal A.

##### Effect of DMSO

No statistically significant differences were found when comparing levels of foci between DMSO treated and untreated cells (Figure 2).

##### Effect of Cal A

**Figure 3:**
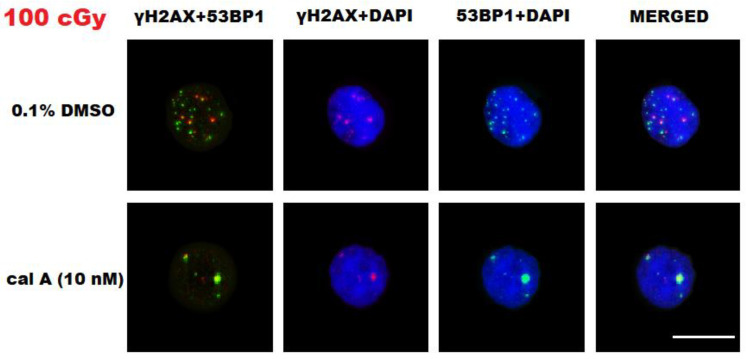
Representative images of DNA repair foci in human umbilical cord blood lymphocytes (UCBL) irradiated with 100 cGy and fixed 2 h after 0.1% DMSO and cal A (10 nM) treatments. Two middle columns represent nuclei (DAPI, blue channel) with γH2AX foci (red channel) and 53BP1 foci (green channel). All three channels (blue, red, and green) are merged in the very right column, while the very left one shows merged red and green channels. White bar equals 10 µm.

Cal A (1 nM) treatment affected neither type of endogenous DNA repair foci. Similarly, the formation of foci induced by low doses of γ-rays (≤10 cGy) was also not affected by cal A (10 nM) treatment. However, this treatment reduced the number of γH2AX foci induced by 100 cGy (*p* = 0.00032) (Figure 2A). The same effect of cal A (10 nM) reducing the number of IRIF after 100 cGy irradiation has also been seen for 53BP1 (*p* = 0.00000) and colocalized γH2AX/53BP1 foci (*p* = 0.00000) (Figure 2). Irradiation of UCBL with low doses of γ-rays did not lead to 53BP1 and colocalized γH2AX/53BP1 foci induction comparing cal A (10 nM) and DMSO treatments (Figure 2). As it is shown below, cal A (10 nM) treatment induced apoptosis as analyzed by both γH2AX pan-staining and annexin V-FITC/PI assays, which may account for the observed decrease in the number of all types of DNA repair foci induced by a high dose of radiation, which effectively induced apoptosis (Figure 3).

**Figure 4:**
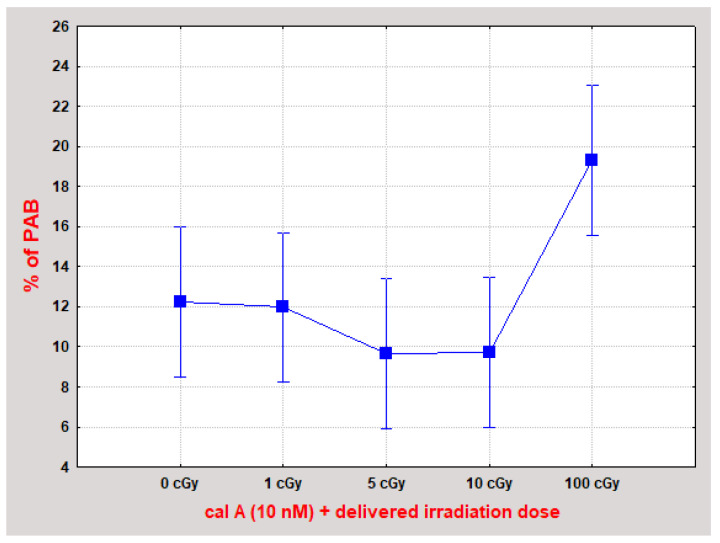
UCBL with pre-apoptotic bodies (PAB) 2 h after irradiation combined with cal A (10 nM) treatment. The combination of cal A (10 nM) + 100 cGy induced statistically significantly more PAB than all other treatments (*p* ˂ 0.01395). Each data point shows the mean value and ±0.95 confidence interval from 3 experiments (at least 200 cells were analyzed in each).

In summary of the evaluated data, we did not observe stabilization of γH2AX foci 2 h post-irradiation by 1 and 10 nM cal A treatment. We have seen a statistically significant decrease in the number of all types of DNA repair foci caused by cal A (10 nM) treatment in 100 cGy irradiated cells. One possible explanation of this effect is that 10 nM cal A combined with a relatively high dose of 100 cGy induces apoptosis-related changes in the cell nucleus structure that prevent DNA damage-binding proteins from accessing the DSB sites. This explanation is supported by the fact that statistically significantly (*p* ˂ A0.01395) (Figure 4), more cells with pre-apoptotic bodies (PAB) were observed after combined treatment with 10 nM cal A and 100 cGy than after treatment of 10 nM cal A combined with lower doses.

#### 2.3.2. Apoptotic Response in UCBL

##### γH2AX Pan-Staining Assay

Apoptosis was assessed by enumerating γH2AX pan-staining, which is usually observed at the early stage of apoptosis [25,26,28,29] but also has been detected in the fraction of late apoptotic cells [28]. Analysis of all data for γH2AX pan-staining has shown its significant dependence on the type of performed treatment (*p* = 0.00004) (Table 1) 2 h post-irradiation, comparing untreated, DMSO-treated cells and both cal A treatments. Univariate one-way ANOVA test showed statistically significant dependence of γH2AX pan-staining on the concentration of cal A (*p* = 0.03556). No dependence on the radiation dose of this apoptotic marker has been observed considering all treatments (*p* = 0.56980) (Table 1). As shown in Figure 2D, irradiation of untreated UCBL to γ-rays did not induce γH2AX pan-staining as 2 h represents too short a time for setting up radiation-induced apoptosis. Similarly, DMSO has had no effect on the level of apoptotic cells detected by γH2AX pan-staining 2 h after irradiation when compared to untreated cells (Figure 2D). While Cal A (1 nM) treatment did not induce γH2AX pan-staining 2 h post-irradiation (Figure 2D), the ratio of γH2AX pan-stained cells was significantly increased by cal A (10 nM) (*p* = 0.022) (Figure 2D). This γH2AX pan-staining induction was seen in between irradiated cal A (10 nM) and irradiated DMSO treated cells, specifically when comparing the doses of 1 and 10 cGy (*p* = 0.04 and 0.03, respectively) (Figure 2D).

Assuming from the obtained data, γH2AX pan-staining assay showed that 2 h treatment of unirradiated/irradiated UCBL with 10 nM cal A forces cells to undergo apoptosis.

##### Annexin V-FITC/PI Assay

**Figure 5:**
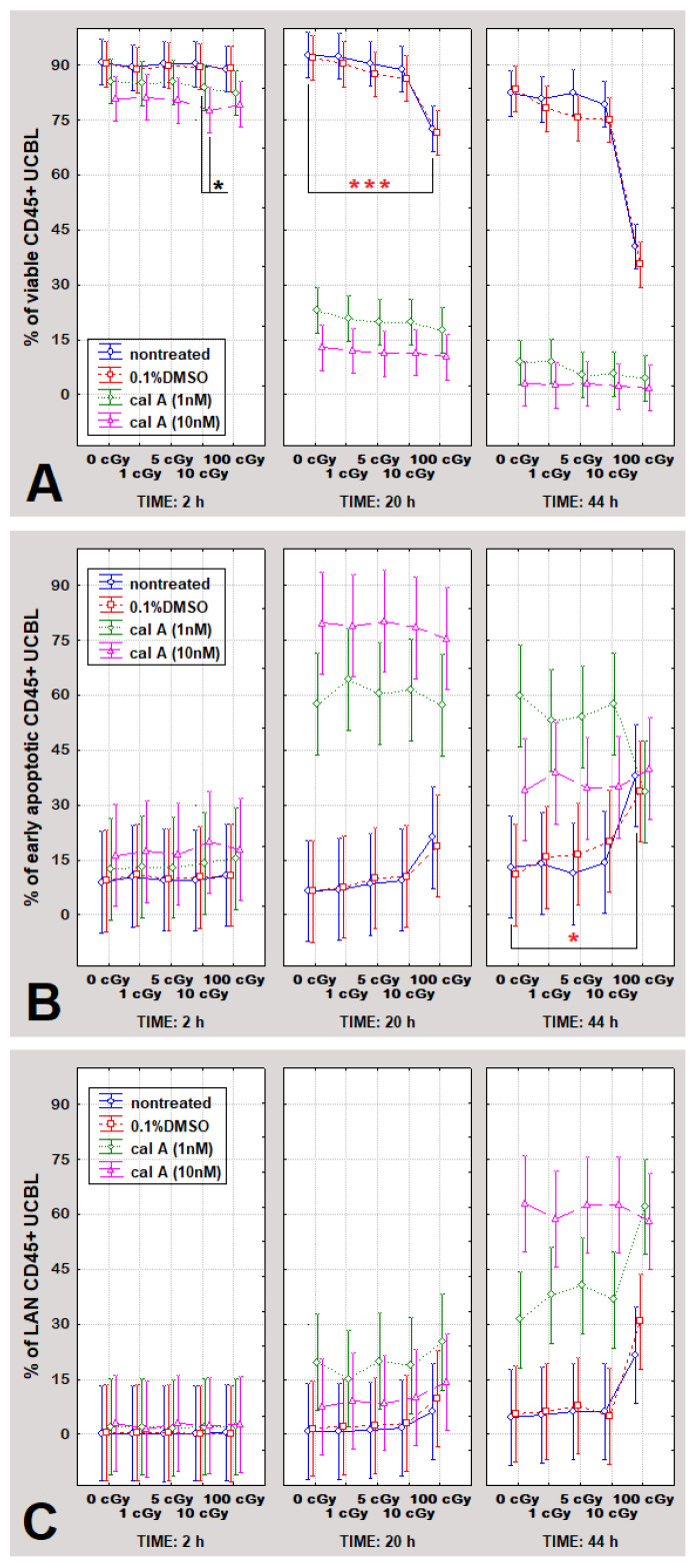
The percentual rates of viable (**A**), early apoptotic (**B**), and late apoptotic/necrotic CD45+ UCBL (**C**) at 2, 20, and 44 h post-irradiation. Cells were treated with 0.1% DMSO, cal A (1 nM), and cal A (10 nM), irradiated with 0, 1, 5, 10, and 100 cGy, and analyzed by the annexin V-FITC/PI assay 2, 20 and 44 h post-irradiation. Each data point shows the mean value and ±0.95 confidence interval from 3 experiments. * indicates statistically significant difference between irradiated and unirradiated cells; * statistically significant differences between treatments. * *p* ˂ 0.05, *** *p* ˂ 0.00099.

Analysis of apoptotic response to different treatment conditions 2 h post-irradiation has further been performed using annexin V-FITC/PI assay, which allows assessing viable, EA, and LAN cells (Figure 5). As shown in Table 1, all pursued apoptotic endpoints responded significantly to the treatment type (*p* ≤ 0.0035). No dependence of all apoptotic endpoints on irradiation dose has been seen 2 h post-irradiation (Table 1). These effects require time to appear to be detected by the flipping of phospholipids in the plasma membrane, which allows annexin V-FITC binding to the cell surface (Figure 5). DMSO-treated cells have not shown any apoptotic changes when compared to untreated UCBL 2 h post-irradiation.

In line with the γH2AX pan-staining data, UCBL treated with 1 nM cal A did not undergo apoptosis as measured with the annexin V-FITC/PI assay. Furthermore, confirming the results obtained by the γH2AX pan-staining assay, a statistically significant decline in viability (*p* = 0.03) was observed in cal A (10 nM) treated cells exposed to 10 cGy contrary to DMSO control irradiated by the same dose (Figure 5A).

### 2.4. Apoptosis in UCBL Induced by Radiation and Cal A 20 and 44 h Post-Irradiation

To further estimate the extent of cal A toxicity, we investigated the induction of apoptosis by cal A in UCBL 20 and 44 h post-irradiation using flow cytometry and annexin V-FITC/PI assay. According to multivariate statistics (ANOVA), all followed apoptotic endpoints (ratios of viable, EA and LAN cells), responded significantly to the type of performed treatment 20 h post-irradiation considering untreated, DMSO-treated cells and both cal A treatments (*p* = 0.00025), but did not depend on delivered radiation doses (Table 2). At 44 h, all apoptotic endpoints depended significantly on both factors (treatment type and dose of radiation). In contrast, the effect of radiation doses on apoptosis was more pronounced (Table 2, Figure 5). When all apoptotic endpoints were considered 2, 20 and 44 h post-irradiation, no statistically significant dependence on cal A concentration occurred, which suggests both 1 nM and 10 nM cal A having a similar apoptotic impact.

#### 2.4.1. Gamma-Radiation

In agreement with literature data [30,31], the dose of 100 cGy induced apoptosis as proven by cell viability decline 20 h after irradiation and considerable increase of EA cells at 44 h post-irradiation (Figure 5). Furthermore, in line with previous studies [32,33], the effect of low radiation doses on apoptosis of UCBL was not detected (Figure 5).

#### 2.4.2. Effect of DMSO

Solely DMSO-treated cells had the same apoptotic response at 20 and 44 h as untreated cells. Similarly, when considering DMSO treatment combined with radiation, DMSO treated cells did not differ in apoptotic response from DMSO untreated ones (Figure 5), meaning that DMSO at this particular concentration itself does not induce apoptosis in UCBL.

#### 2.4.3. Effect of Cal A

Induction of apoptosis in UCBL treated with 10 nM cal A has already been seen 2 h post-irradiation as detected both by γH2AX pan-staining and annexin V-FITC/PI assay. At this time point, consistently less viable cells were spotted in 1 nM cal A treated cells than in DMSO-treated ones (Figure 5). 20 h post-irradiation, the toxicity of 1 nM cal A also became statically significant; however, 10 nM cal A treated cells were forced to undergo apoptosis to a much greater extent than cells treated with 1 nM cal A (Figure 5). Thus, the trend of cal A toxicity revealed already 2 h post-irradiation for 10 nM cal A treatment continued severely for both cal A concentrations up to 20 h post-irradiation when 75–90% of cal A-treated cells became apoptotic. At 44 h, nearly all cal A-treated cells underwent apoptosis (Figure 5).

### 2.5. Correlation Analysis of Apoptotic Response and DNA Repair Focus Formation 2 h Post-Irradiation

Taking all types of the treatments (including both cal A treatments, DMSO treatment and untreated cells) into consideration, a negative correlation (Spearman ROC = −0.288, *p* = 0.02529) was found between the percentage of viable UCBL and the level of γH2AX foci occurrence. Additionally, percentage of early apoptotic cells correlated positively with the level of γH2AX foci (Spearman ROC = 0.316, *p* = 0.01369). No such correlations were spotted for 53BP1 and colocalized foci. When data from each treatment evaluated individually, no correlations were found between all types of foci and γH2AX pan-staining. Considering all treatments, DNA repair foci did not correlate with late apoptosis as defined by the PI uptake and also with γH2AX pan-stained cell incidence. γH2AX pan-staining correlated positively with percentages of early apoptotic and LAN cells (Spearman ROC = 0.692 and 0.273, *p* = 0.00000 and 0.03458, respectively) and negatively with portion of viable cells (Spearman ROC = -0.661, *p* = 0.00000) providing evidence that pan-staining is a reliable endpoint for evaluation of apoptosis. A strong statistically significant positive correlation (Spearman ROC = 0.753, *p* = 0.00006) arose between the ratio of pan-staining and the fraction of LAN cells when 10 nM cal A treatment data were processed. All three types of DNA repair foci strongly positively correlated (Spearman ROC ≥ 0.768), showing that all these endpoints represent valuable molecular markers for DSB.

## 3. Discussion

DNA repair focus assay is considered the most sensitive among the methods used to analyze DNA DSB. The most commonly used biomarkers of DSB are phosphorylated forms of histone H2AX (γH2AX) [34] and 53 binding proteins 1 (53BP1) [35]. Phosphorylation of the histone variant H2AX is one of the earliest cell responses to induction of DSB. The peak number of γH2AX foci is usually observed at 15 min and gradually declines afterward. Rapid kinetics of γH2AX foci dephosphorylation (and thus also of its microscopic visualization) limits the number of the samples that can be managed during one experiment at the time of the peak number focus appearance. It has previously been reported by Kuefner et al. that the γH2AX foci stabilization could be reached by incorporating cal A treatment into the experimental protocol [20]. In this study, cal A inhibited dephosphorylation of γH2AX foci, and thus allowed visibility of the peak-formed γH2AX foci even 2 h post-irradiation, suggesting a methodological approach for analysis of peak number of γH2AX foci in multiple samples by stabilizing degradation of γH2AX foci using cal A. We aimed to replicate these data by analyzing whether cal A dephosphorylation inhibitory features can hold the number of initially accumulated γH2AX even 2 h after irradiation with low (1, 5 cGy and 10 cGy) and high (100 cGy) doses. We also wondered what the cal A effect would be on radiation-induced 53BP1 foci, which were not expected to be influenced. Furthermore, the kinetics of apoptosis was examined 2, 20 and 44 h post-irradiation to assess cal A toxicity.

### 3.1. Gamma-Radiation

In our study, the threshold of detection for γH2AX and 53BP1 foci at 2 h post-irradiation was 10 cGy. Using γH2AX/53BP1 colocalization as an endpoint, the dose detection threshold was just 5 cGy. Our data align with a study where 5 cGy was the lowest detectable dose at 2 h post-irradiation [36]. This is remarkable sensitivity given that the assessment was performed at 2 h post-irradiation when most DSB have been repaired, showing that colocalization of two molecular markers provides better sensitivity to low-dose-induced DSB than each of them alone. Therefore, the sensitivity of the DNA repair focus assay used in our study was comparable with the previously reported data and thus provides reliable information about IRIF induced in human lymphocytes by ionizing radiation.

In our present and in some previous studies, a linear correlation between γH2AX foci and radiation dose was described 2 h post-irradiation with doses less than 100 cGy [36,37,38,39]. However, other studies did not observe this relationship [19]. While the reason for these discrepancies remains unclear, our data provide further evidence for linear dose-response of γH2AX, 53BP1, and their colocalization in human lymphocytes. Therefore, these IRIF molecular markers represent a strong biodosimetry tool for the estimation of low-dose irradiation.

Apoptosis may alter the number of visualized foci due to limited access of DNA markers to the DSB site. This was why we decided to analyze apoptosis along with foci to assess the effects of cal A. Apoptosis is usually assessed by annexin V-FITC/PI assay, but γH2AX pan-staining has also been used to this aim [26,27,28,40]. In line with previous studies [30,31], which reported that human lymphocytes undergo apoptosis within 4–24 h after irradiation in a dose-dependent manner, we did not observe the effect of irradiation on apoptosis with any assay 2 h post-irradiation. The effect of low doses (1, 5 and 10 cGy) on apoptosis was not detected at neither time point, which is in line with previously reported data by Durdik et al. [32] and also Kosik et al. [33] and does not allow using these apoptotic assays for biodosimetry of low doses. No dose dependence of γH2AX pan-staining has been observed considering all treatments, which agrees with the results of Jakl et al. [36]. We reached similar dose detection thresholds in accordance with previously reported data as assessed by the annexin V-FITC/PI assay. Therefore, it represents reliable information about apoptosis induced in human lymphocytes by ionizing radiation.

### 3.2. Effect of DMSO

DMSO is a potent radical scavenger, which is long known as a radioprotector at concentrations above 0.5% [41,42]. In our study, we used 0.1% DMSO, and we did not observe its effect on all types of studied here DNA repair foci. 0.1% DMSO also had no effect on induction of apoptosis as detected by γH2AX pan-staining what was also proven by annexin V-FITC/PI assay 20 and 44 h post-irradiation. Overall, a comparison of time kinetics for apoptotic response between DMSO-treated and untreated UCBL did not reveal any differences even when DMSO was combined with radiation. Therefore, 0.1% DMSO had no effect on human lymphocytes and was a suitable solvent for cal A in our study.

### 3.3. Effect of Cal A

The molecular mechanisms of the disappearance of γH2AX foci at the DSB sites are not completely understood yet. Still, direct dephosphorylation or proteolysis of γH2AX foci in chromatin by protein phosphatases has been suggested [21]. Cal A is a well-known inhibitor of protein phosphatase 1 and 2A [21]. Inhibition of histone γH2AX dephosphorylation by cal A may represent a very useful tool to mark the induced DSB in a permanent way [22]. However, contradictory results on this issue have so far been obtained, as summarized in Table 3. Cal A concentrations of 1 and 10 nM were sufficient to inhibit the decrease of γH2AX foci after irradiation of lymphocytes with low X-ray doses, as reported previously by Kuefner et al. [20]. Stabilization of γH2AX foci in primary human lung fibroblasts irradiated with 100 cGy of γ-rays 2 h post-irradiation by cal A at the concentration of 2.5 nM has also been reported by Antonelli et al. [22]. Nazarov et al. [21] reported that the γH2AX foci could be maintained 6 h post-γ-irradiation with 20 Gy in human A431 cells treated by 10 nM cal A. In our study, the usage of cal A treatment did not enable maintenance of the peak γH2AX foci value in irradiated lymphocytes 2 h post-irradiation. Lack of cal A effect on γH2AX focus stabilization was also reported by Roch-Lefevre et al. [24] and Jakl et al. [23].

Especially dramatic differences were observed between Kuefner et al. study [20] from one side and Roch-Lefevre et al. [24], Jakl et al. [23] and our study, from another, given that all these studies were performed with human lymphocytes and the same time point, 2 h post-irradiation, was used for assessment. Frozen cells were used in our and Jakl et al. study. In contrast, Kuefner et al. used freshly harvested lymphocytes. On the other hand, Roch-Lefevre et al. [24] also reported unsuccessful stabilization of peak γH2AX foci using fresh peripheral blood lymphocytes (Table 3). Thus, freezing–thawing cycle does not look like a major reason for the contradictory effects observed in available studies (Table 3). We assume that the reported variability may be caused by the interplay between ongoing apoptosis and γH2AX focus formation. In our study, cal A-induced apoptosis already 2 h post-irradiation. This cal A-induced apoptosis might affect γH2AX formation by inhibiting phosphorylation of γH2AX or preventing DNA repair focus formation due to apoptosis-related chromatin changes. This assumption is supported by the data showing that cal A treatment significantly decreased number of both γH2AX and 53PB1 foci induced by 100 cGy. Indeed, about 2-fold less 53BP1 as well as colocalized γH2AX/53BP1 IRIF were observed in cal A (10 nM) treated and irradiated with 100 cGy 2 h post-irradiation compared with the cal A-untreated, but irradiated cells. Radiation induced γH2AX foci were also inhibited by this cal A treatment.

No data on apoptosis induced in lymphocytes by radiation and cal A were reported by Kuefner et al. to be compared with our data.

**Figure 6:**
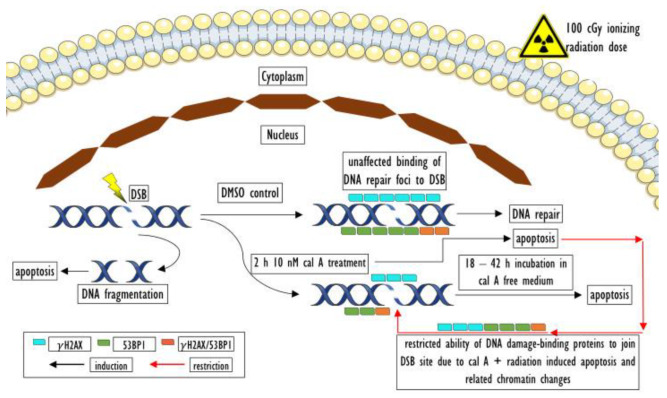
Possible explanation of low DNA repair foci level in 10 nM cal A + 100 cGy treated UCBL 2 h posttreatment. Our assumption is that cal A + 100 cGy irradiation-induced apoptosis and related changes in chromatin structure prevent DNA damage-binding proteins from accessing the DSB site. Thus, lower numbers of DNA DSB repair foci could be detected in irradiated and cal A 10 nM treated cells than in irradiated cells.

As we have already suggested, apoptosis induced by cal A 10 nM may account for the decrease in the number of all types of DNA repair foci 2 h post-irradiation. We assume that induction of apoptosis mediated by cal A and 100 cGy has led to changes in the structure of nucleus/chromatin. This assumption is supported by the fact that significantly more cells with PAB were observed after the combined 10 nM cal A + 100 cGy treatment than after treatment of 10 nM cal A combined with lower doses. Apoptosis has been shown to lead to chromatin changes in various mammalian cells [43]. Cal A triggered apoptosis may then restrict DNA damage repair proteins from accessing the DSB site. Thus, lower numbers of DSB markers could have been detected in cal A 10 nM + 100 cGy-treated cells than in irradiated cells (Figure 6). As no such inhibition of DNA repair foci occurred in cal A 1 nM + 100 cGy-treated cells, we can conclude that toxicity of cal A (also combined with a relatively high dose of γ-rays) in human lymphocytes grows hand-in-hand with its increasing concentration.

Cal A 10 nM treatment has induced γH2AX pan-staining 2 h post-irradiation, while 1 nM cal A has not. These observations on dose-dependent induction of apoptosis by cal A were also confirmed by annexin V-FITC/PI assay. This outcome is in line with data reported by Nakamura and Antoku that treatment with 10 nM cal A enhanced radiation-dependent killing of cultured mammalian cells [44]. As far as cal A failed to maintain γH2AX foci in our study, we assume that γH2AX pan-staining induced by 10 nM cal is not caused by inhibition of γH2AX dephosphorylation, but rather is a direct result of cal A toxicity. This assumption was supported by a statistically significant positive correlation found between the ratio of pan-staining and the fraction of early apoptotic cells (analyzed by annexin V-FITC/PI assay) considering all performed treatments. In addition, a strong statistically significant positive correlation arose between the ratio of pan-staining and the fraction of LAN cells when just cal A 10 nM data were statistically processed. These findings support previously reported data that γH2AX pan-staining occurs in both EA and LAN cells [28].

Assuming from the obtained data, both assays used to assess apoptosis confirmed that cal A treatment induces apoptosis of UCBL 2 h posttreatment, thus uncovered its toxicity and UCBL sensitivity to this agent. Enumeration of γH2AX pan-staining by fluorescent microscopy seems to represent a method similar in its sensitivity to annexin V-FITC/PI assay in the evaluation of apoptosis. The trend of cal A toxicity, which was revealed already 2 h post-irradiation, continued severely for both cal A concentrations almost evenly up to a time point of 44 h when nearly all cells have already undergone apoptosis. Apparently, apoptosis triggered by cal A in UCBL may underlie the failure of cal A to maintain γH2AX IRIF.

## 4. Materials and Methods

### 4.1. Chemicals

Reagent grade chemicals were obtained from Sigma-Aldrich (St. Louis, MO, USA), Merck KgaA (Darmstadt, Germany) and Life Technologies (Carlsbad, CA, USA).

### 4.2. Umbilical Cord Blood Cells

This study was approved by the Ethics Committee of Children’s Hospital in Bratislava. Umbilical cord blood mononuclear cells (UCB MNC) were extracted as previously described [25] from three healthy newborns after full-term pregnancies and provided by Dr. M. Kubes, Eurocord-Slovakia, Bratislava, Slovak Republic.

Frozen UCB MNC were thawed and diluted in 10 mL of thawing medium containing 4.5 mL of Hank’s balanced salt solution (HBSS) medium (Gibco, Life Technologies, Cramlington, UK), 1 mL of 1 mg/mL DNAse I (Sigma-Aldrich (St. Louis, MO, USA), (100 μg/mL working concentration) and 4.5 mL of Roswell Park Memorial Institute (RPMI) 1640 medium with L-glutamine and 4-(2-hydroxyethyl)-1-piperazineethane-sulfonic acid (HEPES) (PAA Laboratories GmbH, Pasching, Austria). Adherent cells (monocytes) were excluded after 2 h incubation of cells in 25 mL of basal medium (BM): RPMI 1640 medium, supplemented with 10% fetal bovine serum (FBS), 100 IU/mL penicillin, 100 mg/mL streptomycin (Gibco, Invitrogen, Karlsruhe, Germany). The viability of remaining umbilical cord blood lymphocytes (UCBL) was not less than 95%, as defined by the Trypan blue exclusion assay.

### 4.3. Cell Treatment

After 2 h incubation, UCBL were spun down at 100 g, diluted in 50 mL (approximate cell density of 2 × 10^6^/mL) of BM and then divided into 4 aliquots (12.5 mL) for subsequent treatments with calyculin A (cal A) (ab 141,784) (Abcam Biochemicals®, Cambridge, UK) dissolved in dimethyl sulfoxide (DMSO) (SERVA Electrophoresis GmbH, Heidelberg, Germany) to stock solutions of 1 and 10 μM to get final concentrations of cal A in cell suspensions of 1 and 10 nM (1) cal A (1 nM) and (2) cal A (10 nM); (3) with 0.1% DMSO (same concentration of DMSO as used in cal A treatments); (4) untreated cells serving as a control to treatments mentioned above. Each of these 4 differently treated UCBL cell suspensions (cal A (1 nM), cal A (10 nM), 0.1% DMSO, untreated) was immediately divided into 5 aliquots of 2.5 mL and incubated on ice 40 min before irradiation.

### 4.4. Irradiation

UCBL were irradiated by γ-rays at the dose rate of 0.45 Gy/min with doses 1, 5, 10, and 100 cGy using cobalt ^60^Co-TERAGAM^®^ K-02 source (UJP Praha, Prague, Czech Republic). The dose deviation was below 5%. The irradiated cells were immediately warmed to 37 °C (±0.1 °C) in a water bath (ED (v.2), Julabo Labortechnik GmbH, Seelbach, Germany) and then incubated at 37 °C in a humidified incubator (NB-203XL, N-BIOTEK, Inc., Bucheon, South Korea) at 5% CO_2_ before fixation.

### 4.5. Immunofluorescence and Image Acquisition

At 2 h post-irradiation, UCBL were washed with phosphate-buffered saline (PBS, 3.2 mM Na_2_HPO_4_, 0.5 mM KH_2_PO_4_, 1.3 mM KCl, 135 mM NaCl, pH 7.4) and spun down using Shandon double Cytofunnels (Thermo Scientific, Runcorn, Cheshire, UK) on the double cytoslides coated with polysine (Thermo Scientific, Menzel-Glaser, Braunschweig, Germany) at 85 g, 5 min using Cellspin I cytocentrifuge (Tharmac GmbH, Tuttlingen, Germany). The cells were fixed with 3% paraformaldehyde for 15 min and left in the fridge overnight (for 16–17 h). Cells were then permeabilized with 0.2% TRITON X-100 for 5 min, washed extensively with PBS and blocked in 3% FBS (Gibco, Germany) for 30 min at room temperature (RT).

The primary antibodies, monoclonal γH2AX anti-mouse (cat. no. #NB100-78356, Novus Biologicals, UK) (dilution 1:800) and polyclonal 53BP1 anti-rabbit (cat. no. #NB100-304, Novus Biologicals, UK) (dilution 1:1000), were diluted in 3% FBS in PBS and applied in 100 µL aliquots to the slides. The slides were incubated for 1 h in a humidified chamber at RT. After washing with PBS, the secondary antibodies, Alexa Fluor 488 IgG (H+L) polyclonal anti-rabbit (cat. no. #A11029, Invitrogen Molecular Probes, Life Technologies, USA) (dilution 1:200) and Alexa Fluor 555 IgG (H+L) monoclonal anti-mouse (cat. no. 21429, Invitrogen Molecular Probes, Life Technologies, USA) (dilution 1:200), were added. Then, the slides were incubated for 1 h in the humidified chamber at RT, washed with PBS, and counterstained with antifade reagent agent VECTASHIELD (Vector Laboratories, Peterborough, UK) containing 4′,6-diamidino-2-phenylindole (DAPI).

Image acquisition of DSB repair foci was conducted using the Metafer slide scanning system V 3.9 (MetaSystems GmbH, Altlussheim, Germany) and Zeiss Axio Imager. Z2 epifluorescent microscope (Carl Zeiss Microscopy GmbH, Gottingen, Germany). The main parameters of image acquisition were: 63x objective magnification, 11 focus planes, and 28/40 μm focus plane distance. Acquired images have been evaluated automatically as previously described [26]. γH2AX/53BP1 foci were considered to colocalize when both types of foci met the requirements of being counted as actual foci, and the distance between their brightest spots did not exceed 0.5 μm. We have also quantified γH2AX pan-stained cells that exhibit whole nuclear γH2AX staining, which occurs in the early stages of apoptosis. γH2AX pan-stained cells were analyzed simultaneously with foci enumeration.

### 4.6. Flow Cytometry

UCBL were harvested at 2, 20, and 44 h after irradiation (the medium containing cal A was changed for the fresh one without cal A at 2 h post-irradiation), spun down (100 g/10 min), washed with PBS and resuspended in 100 µL of annexin kit buffer (Roche, Basel, Switzerland). Cells were then stained with the antibody against the cell surface markers: anti-human CD45-APC clone: 5B1 (isotype: mouse IgG2a) (cat. no. 130–091–230, Miltenyi Biotec GmbH, Bergisch Gladbach, Germany) to determine lymphocyte population, annexin V-FITC (BD Biosciences, San Jose, CA, USA) and propidium iodide (PI) (BD Biosciences). Samples were then incubated for 20 min in the dark at RT, washed with PBS, spun down, diluted in 200 µL of annexin kit buffer and analyzed by the BD Accuri C6 flow cytometer (Accuri Cytometers, Inc., Ann Arbor, MI, USA). Lymphocytes (CD45-APC-positive cells) were analyzed on the annexin V-FITC/PI scatter and the percentage of early apoptotic (EA) (annexin V-FITC positive/PI-negative) and late apoptotic/necrotic (LAN) cells (annexin V-FITC positive/PI-positive) was determined.

### 4.7. Statistical Analysis

The data were analyzed by the factorial analysis of variance (ANOVA) and the false discovery rate (FDR). The comparison between groups for pooled data that fitted normal distribution (analyzing designs with a single categorical independent variable) was performed using a one-way ANOVA test, adjusted by post hoc Fisher’s LSD test. Correlations between groups were computed using the Spearman rank-order correlations test (ROC). All statistical operations were carried out using Statistica 8.0 software (StatSoft Inc., Tulsa, OK, USA). The results were considered significantly different at *p* < 0.05. The radiation-induced excess over unirradiated control (IRIF) observed after irradiation was approximated by linear regression Y = ßD, validated by the coefficient of determination R^2^.

## 5. Conclusions

The usage of cal A treatment in our study did not enable maintaining the peak value of γH2AX foci in irradiated lymphocytes 2 h post-irradiation. However, the opposite result has previously been reported. We assume that this different outcome could have been caused by cal A-induced apoptosis resulting in (i) inhibition of H2AX phosphorylation, or/and (ii) changes in nucleus/chromatin structure observed in our study and supported by both negative correlations between the number of γH2AX foci and cell viability decline and positive correlation identified between rate of EA cells and γH2AX foci appearance. Both assays used for assessment of apoptosis confirmed that 10 nM cal A treatment induces apoptosis of UCBL 2 h posttreatment independently from delivered radiation dose. We assume that cal A-induced apoptosis may stand behind failure to maintain radiation-induced γH2AX foci and may underlie the observed reduction of γH2AX/53BP1 foci induced by high-dose radiation. All DSB molecular markers used in this study responded in a linear manner to low doses of ionizing radiation. Therefore, their combination may represent a strong biodosimetry tool for estimation of radiation response to low doses. Assessment of colocalized γH2AX/53BP1 improves the threshold of low dose detection.

## Figures and Tables

**Table 1:** Statistical testing dependence of DNA repair foci and apoptotic endpoints on categorical factors: radiation dose (Dose) and cal A/DMSO treatments (Treatment) 2 h post-irradiation.

Variables							Multifactorial ANOVA		Categorical Factors		*p*-Values Adjusted by FDR	
γH2AX *	53BP1 *	γH2AX/ 53BP1 *	Viable (%)	EA (%)	LAN (%)	Pan-st. (%)	Multivariate	Univariate	Dose	Treatment	Dose	Treatment
+	+	+	+	+	+	+	+	-	+	+	0.00000	0.00000
+	-	-	-	-	-	-	-	+	+	+	0.00000	0.36717
-	+	-	-	-	-	-	-	+	+	+	0.00000	0.00000
-	-	+	-	-	-	-	-	+	+	+	0.00000	0.00000
-	-	-	+	-	-	-	-	+	+	+	0.95145	0.00032
-	-	-	-	+	-	-	-	+	+	+	0.91988	0.00241
-	-	-	-	-	+	-	-	+	+	+	0.98870	0.00359
-	-	-	-	-	-	+	-	+	+	+	0.56980	0.00004

* mean, foci per cell; + the data were included in analysis; - data were not included in analysis. Statistically significant values are marked red.

**Table 2:** Statistical testing dependence of all apoptotic endpoints on categorical factors: radiation dose (dose) and cal A/DMSO treatments (treatment) 2, 20, and 44 h post-irradiation.

Variables			Multifactorial ANOVA		Categorical Factors			*p*-Values Adjusted by FDR		
Viable (%)	EA (%)	LAN (%)	Multivariate	Univariate	Time	Dose	Treatment	Time	Dose	Treatment
+	+	+	+	-	all	+	+	0.00000	0.00000	0.00000
+	-	-	-	+	all	+	+	0.00000	0.00000	0.00000
-	+	-	-	+	all	+	+	0.00000	0.51274	0.00000
-	-	+	-	+	all	+	+	0.00000	0.02060	0.00000
+	+	+	+	-	2 h	+	+	-	0.82175	0.00485
+	-	-	-	+	2 h	+	+	-	0.95145	0.00039
-	+	-	-	+	2 h	+	+	-	0.91988	0.00241
-	-	+	-	+	2 h	+	+	-	0.98870	0.00359
+	+	+	+	-	20 h	+	+	-	0.77601	0.00025
+	-	-	-	+	20 h	+	+	-	0.92268	0.00000
-	+	-	-	+	20 h	+	+	-	0.99875	0.00002
-	-	+	-	+	20 h	+	+	-	0.84090	0.09587
+	+	+	+	-	44 h	+	+	-	0.00000	0.00000
+	-	-	-	+	44 h	+	+	-	0.00000	0.00000
-	+	-	-	+	44 h	+	+	-	0.77013	0.00000
-	-	+	-	+	44 h	+	+	-	0.04194	0.00000

+ the data were included in analysis; - data were not included in analysis. Statistically significant values are marked red.

**Table 3:** Methodological information on studies investigating the effect of cal A on radiation-induced γH2AX foci.

Author	Cell Type	Solvent of Cal A	Concentration of Cal A (nM)	Cell Treatmet with Cal A Prior-/Post-Irradiation	Dose of Irradiation (Gy)	Effect on γH2AX Foci	Analysis of Apoptosis
Nazarov et al. (2003)	Chinese hamster V79	unknown	10	post-; for 0.5 h	20	stabilization identified at 6 h	NO
Antonelli et al. (2005)	Primary human lung fibroblasts (MRC-5)	unknown	2.5	post-; continual cal A treatment	1	stabilization identified at 2 h	NO
Roch-Lefevre et al. (2010)	Human peripheral blood lymphocytes	unknown	5	prior-; whole blood irr.; continual cal A treatment	0.05–5	no stabilization identified at 2 h	YES
Kuefner et al. (2013)	Human peripheral blood lymphocytes	ethanol	1; 10	prior-; continual cal A treatment	0.001–0.01	stabilization identified at 2 h	colony formation assay (but not using lymphocytes)
Jakl et al. (2016)	Human umbilical cord blood lymphocytes	DMSO	1; 10	immediately prior-/post; continual for 2 h	0.001–0.01	no stabilization identified at 2 h	NO

## Data Availability

Not applicable.

## Abbreviations

BM	Basal medium
cal A	Calyculin A
CFA	Colony formation assay
CT	Computed tomography
DAPI	4′,6-diamidino-2-phenylindole
DMSO	Dimethyl sulfoxide
DNA DSB	DNA double-strand breaks
EA cells	Early apoptotic cells
FDR	False discovery rate
HBSS	Hanks’ balanced salt solution medium
HEPES	4-(2-hydroxyethyl)-1-piperazineethane-sulfonic acid
IRIF	Ionizing radiation-induced foci
LAN cells	Late apoptotic/necrotic cells
PAB	Pre-apoptotic bodies
PI	Propidium iodide
RPMI	Roswell Park Memorial Institute medium
RT	Room temperature
Spearman ROC	Spearman rank-order correlations test
UCB MNC	Umbilical cord blood mononuclear cells
UCBL	Umbilical cord blood lymphocytes
